# Novel *HCN1* Mutations Associated With Epilepsy and Impacts on Neuronal Excitability

**DOI:** 10.3389/fnmol.2022.870182

**Published:** 2022-06-30

**Authors:** Changning Xie, Fangyun Liu, Hailan He, Fang He, Leilei Mao, Xiaole Wang, Fei Yin, Jing Peng

**Affiliations:** ^1^Department of Pediatrics, Xiangya Hospital, Central South University, Changsha, China; ^2^Hunan Intellectual and Development Disabilities Research Center, Changsha, China

**Keywords:** epilepsy, intellectual disorders, patch clamp, neuronal excitability, *HCN1*

## Abstract

Hyperpolarization-activated cyclic nucleotide-gated (HCN) channel plays a critical role in regulating the resting membrane potential and integrating synaptic transmission. Variants of *HCN1* have been recognized as causes of epilepsy, and mutant HCN1 channels could act with loss-of-function (LOF), loss- and gain-of-function (LOF and GOF) and gain-of-function (GOF) mechanisms. However, phenotypes and pathogenesis of HCN1-related epilepsy are still poorly understood. This study enrolled five epileptic cases carrying five different *HCN1* variants: two pathogenic variants (I380F and S710Rfs*71), two likely pathogenic variants (E240G and A395G), and a paternally inherited variant (V572A). Four variants were novel. Electrophysiological experiments revealed impaired biophysical properties of the identified mutants, including current densities and activation/deactivation kinetics. Moreover, three variants exerted effects on the biophysical properties of wild-type HCN1 channels in heterozygous conditions. Immunofluorescence experiments showed that two variants reduced the protein expression of HCN1channels in neurons. Neurons expressing E240G (GOF) variant showed increased input resistance. However, the variant of I380F (LOF) increased the neuronal firing rate, thus leading to neuronal hyperexcitability. In conclusion, the present study expands the genotypic and phenotypic spectrum of patients with *HCN1*-related epilepsy and clarifies the underlying mechanisms. We reported five new cases including four unreported likely/pathogenic variants. We provided assessments of biophysical function for each variant, which could help patients to receive individual therapy in the future. We confirmed that *HCN1* variants contributed to neuronal hyperexcitability by regulating input resistance and the action potential firing rate, and we have shown that they can affect protein expression in neurons for the first time.

## Introduction

With the development of whole-exon sequencing, mutations in *HCN1* have been identified as causes of epilepsy (Oyrer et al., 2018). Hyperpolarization-activated cyclic nucleotide-gated ion channel 1 (HCN1), which is encoded by the *HCN1* gene, contains an amino-terminal domain, six transmembrane domains (S1–S6), and a carboxyl terminus with a cyclic nucleotide-binding domain (Lee and MacKinnon, 2017). HCN channels are enriched in the brain and play important roles in the resting membrane potential, neuronal rhythmic activity, and dendritic integration (Brewster et al., 2006; Postea and Biel, 2011) by mediating hyperpolarizing activated non-selective cation current (Ih) (Benarroch, 2013; Santoro and Shah, 2020). Initially, the clinical manifestation of *HCN1*-related epilepsy was termed Dravet syndrome (Steel et al., 2017; Mei et al., 2019). Indeed, they have some similarities, such as early onset age, fever sensitivity, and developmental regression (Nava et al., 2014; Wang et al., 2019). Over the years, more phenotypes of *HCN1*-related epilepsy have been identified, including severe epilepsy of infancy with migrating focal seizures, absence, and other neurodevelopmental diseases (Lucariello et al., 2016; Marini et al., 2018). Clinical manifestations including frequency of episodes, severity, and type of seizures vary among patients.

Previous studies have shown variants of *HCN1* have loss-of-function (LOF) and gain-of-function (GOF) effects on HCN1 channels (Nava et al., 2014; Bonzanni et al., 2018; Marini et al., 2018; Porro et al., 2021). LOF variants impact neuronal excitability, including resting membrane potential, input resistance, and firing properties (Bonzanni et al., 2018; Bleakley et al., 2021), contributing to epilepsy. However, the altered functions of the mutated channels are various, including current densities, voltage-dependent activation, and/or altered kinetics. There is no doubt that this variety brings great challenges to the description of the relationship between the function of mutated channels and clinical phenotypes, as well as neuronal mechanisms underlying epilepsy.

In the present study, we reported five new patients with *HCN1*-related epilepsy and identified four unreported variants. We found all five variants showed altered biophysical properties of HCN1 channels. Furthermore, neurons expressing mutant channels showed reduced HCN1 protein expression and impaired neuronal excitability.

## Materials and Methods

### Patient Recruitment

This study was approved by the ethics committee of Xiangya Hospital of Central South University. Written informed consent was obtained from parents or guardians of all patients before any study. We retrospectively collected clinical data from patients, including seizure onset, seizure types, electroencephalogram (EEG), brain MRI, and anti-seizure therapy. All EEG recordings were obtained with a time-locked synchronized video and carefully analyzed by a certified pediatric neurologist and two Asian Epilepsy Academy certified electroencephalographers. Genetic analysis was performed by trio whole-exome sequencing, and candidate causative variants were confirmed by Sanger sequencing. We rechecked all the variants with nucleotide and amino acid numbering according to the *HCN1* reference transcript (NM_021072). The minor allele frequency of variants and pathogenicity were evaluated using Genome Aggregation Database (gnomAD)^1^ and two missense prediction programs (SIFT, PROVEAN). The clinical significance of variants was determined under the American College of Medical Genetics and Genomics (ACMG) (Richards et al., 2015) standard guidelines.

### Cell Culture and Transfection

Human embryonic kidney (HEK) 293 cells were cultured in Dulbecco’s Modified Eagle’s Medium (supplemented with 10% FBS and 1% penicillin/streptomycin). The human *HCN1* plasmids were purchased from Genscript Corporation. *HCN1* cDNA was subcloned into the pCDNA3.1 vector or pCAGGS vector for neurons, and variants were introduced into this cDNA with the KOD site-directed mutagenesis kit (Toyobo). Then, correct constructs were confirmed by sequencing and reserved for subsequent experiments. The mixtures of WT or mutant *HCN1* plasmids (1 μg) and 0.5 μg of enhanced fluorescent protein (EGFP) were transfected into HEK 293 cells by Lipofectamine (Invitrogen). For co-expression experiments, WT plasmids, mutant *HCN1* plasmids, and EGFP (at a ratio of 1:1:0.5) were mixed and added to the cultures.

Primary cortical neurons were obtained from embryonic day 17–18 (E17-18) C57BL/6 mice as previously reported (Guo et al., 2019). Briefly, the fetal cortex was digested by adding 2 mg/ml papain (Worthington Biomedical Corporation) and incubating for 30 min at 37°C. Neurons were seeded onto 12-mm diameter coverslips at a density of 10^5^ cells/well in the culture medium (neurobasal medium supplemented with 2% B27, 1% glutamax, and 1% penicillin/streptomycin). One microgram of WT/mutant plasmids and 0.25 μg of enhanced GFP DNA were transfected using lipofectamine at day *in vitro* 8–9 (DIV8-9).

### Electrophysiology

Electrophysiological recordings were performed as described in the previous studies (Marini et al., 2018). Briefly, experiments were performed on CHO cells 24–36 h after transfection. The pipette solution contained the following compounds (in mM): 120 potassium aspartate, 10 KCl, 10 NaCl, 10 EGTA, 1 CaCl_2_, 10 HEPES, and 2 Mg-ATP. The bath solution contained (in mM) NaCl 130, KCl 15, MgCl_2_ 0.5, CaCl_2_ 1.8, glucose 10, and HEPES 5. To determine the current–voltage relationship, the peak current was measured at various hyperpolarization steps from the holding potential of –20 mV (–10 mV increment, hyperpolarized to –130 mV) and normalized to membrane capacitance. To obtain the activation curves of the HCN1 channels, tail currents were recorded at –110 mV (or –130 mV) after hyperpolarized steps from –20 to –110 (or –130) mV in –10 mV increments and calculated by the Boltzmann function:


I(V)=I/m⁢a⁢x(1+exp(V-V)1/2/k)V

where I is the recorded current amplitude at test potential, I_max_ is the maximal current, V is the voltage, V_1/2_ indicates the voltage at half-maximal activation, and K_v_ is the slope. Activation time constants were obtained by fitting current traces acquired from the activation protocol using a double-exponential function after an initial delay, and deactivation time constants were obtained by fitting current traces recorded (at + 10 mV) after a fully activating step at –120 mV.

All data were acquired using a 700 B amplifier (Molecular Devices) and analyzed using Clampfit 10.6 software (Axon Instruments). The series resistance was < 10 MΩ.

For neurons, current-clamp experiments were performed at DIV 4–5 after transfection. Cells were held at –70 mV, and resting membrane potentials were recorded within 2 min after the membrane breaking (I_holding_ = 0 pA). The intracellular solution is composed of the following compounds (in mM): 123 K-gluconate, 10 KCl, 1 MgCl_2_, 1 EGTA, 0.1 CaCl_2_, 0.2 Na-GTP, 1.5 Mg-ATP, 4 D-glucose, and 10 Hepes (pH = 7.3, 290 mOsm). The extracellular solution contains the following compounds (in mM): 140 NaCl, 3 KCl, 2 CaCl_2_, 1 MgCl_2_, 10 D-glucose, and 10 Hepes. To block excitatory and inhibitory synaptic inputs, APV (20 μM), CNQX (10 μM), and bicuculline (10 μM) were added. The spikes were elicited by current injection ranging from 0 to 180 pA with 20 pA increment, lasting for 500 ms. Input resistance was calculated when cells received a current stimulus (–100 pA). Spike threshold was defined as the first current injection being able to elicit action potentials. Sag ratio was calculated using the equation: Sag ratio = (V_peak_ - V_ss_)/V_peak_, where V_peak_ represents the maximum voltage and Vss indicates the steady-state voltage at the late phase of the hyperpolarizing current step.

### Immunocytochemistry

Cells were fixed with 4% paraformaldehyde in PBS for 10 min. Next, they were blocked with 5% BSA with or without (for surface expression) 0.3% Triton after washing in PBS. They were next incubated with anti-HCN1 antibody (Alomone, 1:500, overnight, 4°C) followed by secondary antibodies (anti-rabbit Cy3, Jackson lab, 6 h, room temperature). Subsequently, the coverslips were washed with PBS for three times and mounted on glass slides. Fluorescent images were acquired using a confocal microscope (Zeiss, LSM800).

### Statistics

Data are represented as mean ± SEM. Statistical analyses were performed using SPSS software, version 17.0 (SPSS Inc., Chicago, IL). Shapiro–Wilk’s test and Levene’s test were used to verify the normality and homogeneity of variance, respectively. Two-group comparisons were carried out using an independent two-sample *t*-test. Comparison of multiple groups was assessed by one-way ANOVA, followed by the Bonferroni *post hoc* test or Dunnett’s T3 test. The Mann–Whitney test was used to evaluate the statistical significance where data were not conformed to normal distribution. In all cases, the significance level was set at *P* < 0.05.

## Results

### Identification of Hyperpolarization-Activated Cyclic Nucleotide-Gated Ion Channel 1 Variants in Five New Cases

**FIGURE 1:**
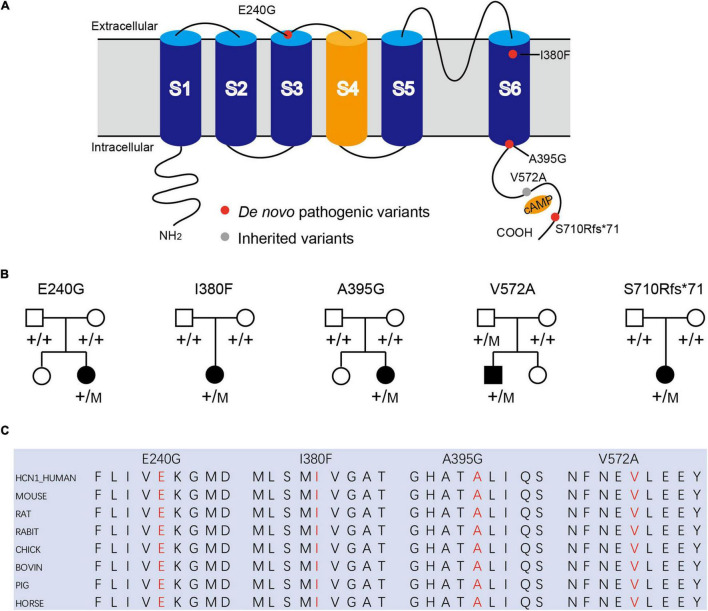
Mutations in *HCN1* channels. **(A)** Structure of HCN1 channels with S1-S6 segments and N/C-terminal region. **(B)** Pedigrees of patients with HCN1 variants. **(C)** All variants are highly conserved among different species.

**FIGURE 2:**
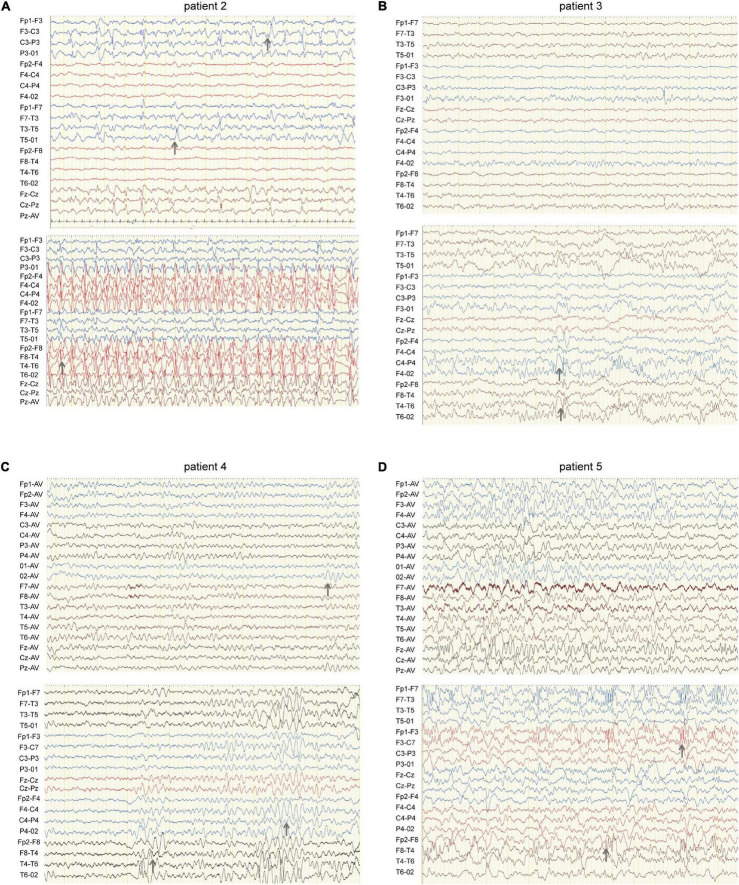
EEG of four patients carrying *HCN1* variants. **(A)** EEG of patient 2 (I380F) shows sharp waves in the left hemisphere during sleep (top) and spike waves during status epilepticus (bottom). Gray arrows indicate abnormal EEG waves. **(B)** EEG of patient 3 (A395G) shows sharp and sharp slow waves in the right occipital region during sleep (bottom). **(C)** EEG of patient 4 (S710Rfs*71) features a slow rhythm of background in the occipital region (top) and a paroxysm of diffused 4–5 Hz slow waves (bottom). **(D)** EEG of patient 5 (V572A) shows θ rhythm mixed δ waves in the background (top) and diffused 3.5–4 Hz multifocal spike-slow waves during a tonic-clonic seizure, while the patient was having a tonic-clonic seizure in sleep (Figure 2D) (bottom).

We identified four novel *HCN1* variants (E240G, A395G, S710Rfs*71, and V572A) and one previously reported variant (I380F) in five patients. All the five variants were classified as likely pathogenic or pathogenic under the ACMG variant classification guidelines (Supplementary Table 1). Clinical and genetic features are presented in Table 1, Figures 1, 2, Supplementary Tables 1, 2, and Supplementary Figure 1.

**TABLE 1:** Phenotypic features of patients with different type of variants according to channel properties.

*N* = 33	LOF (*n* = 18)	GOF(*n* = 13)	GOF/LOF (*n* = 2)
Variants	p.Met243Arg (2); p.Ser272Pro (1); p.Arg297Thr (1); p.Met305Leu (2); p.Cys329Ser (5); p.Ile380Phe (1); p.Gly391Asp (2); p.Gly391Cys (1); p.Ala395Gly (1); p.Ser399Pro (1); p.Arg590Gln (1)	p. Ser100Phe (1); p. Met153Ile (2); p. Glu240Gly (1); p. His279Tyr (1); p. Gly391Ser (2); p. Ile397Leu (1); p. Asp401His (1); p.Val414Met (3); p.Val572Ala (1)	p. Leu157Val; p. S710Rfs*71
Domain	S3–S4 (1/11); S4 (1/11) S5 (2/11); S5–S6 (1/11); S6 (3/11); C (3/11)	N (1/9); S1 (1/9); S3–S4 (1/9); S4 (1/9); S6 (1/9); C (4/9)	S1 (1); C (1)
Epilepsy onset age	< 1 Year (13/18); > 1 year (5/18)	<1 Year (8/13); > 1 year (5/13)	< 1 Year (1/2); > 1 year (1/2)
Seizure types	Focal (4/15)	Focal (7/11)	Focal (1/2)
	Generalized (14/15)	Generalized (11/11)	Generalized (1/2)
	Both (3/15)	Both (7/11)	Both (0/2)
Sensitivity to fever	(12/17)	(12/13)	(1/2)
Status epilepticus	(5/18)	(1/13)	(0/2)
Seizure outcome	Seizure-free without therapy (0/18); seizure-free with monotherapy (6/18); seizure-free with combined therapy (2/18)	Seizure-free without therapy (2/13); seizure-free with monotherapy (4/13); seizure-free with combined therapy (1/13)	Seizure-free without therapy (0/2); seizure-free with monotherapy (2/2); seizure-free with combined therapy (0/2)
	Uncontrolled (10/18)	Uncontrolled (6/13)	Uncontrolled (0/2)
Intellectual disability	Normal (6/18)	Normal (6/13)	Normal (2/2)
	Mild (2/18)	Mild (4/13)	Mild (0/2)
	Moderate/severe (10/18)	Moderate/severe (3/13)	Moderate/severe (0/2)
Language or movement disorders	(7/7)	(5/6)	(0/1)
EEG	Normal (6/15); abnormal (9/15)	Normal (1/12); abnormal (11/12)	Normal (0/2); abnormal (2/2)
MRI	Normal (11/14); abnormal (3/14)	Normal (11/11); abnormal (0/11)	Normal (2/2); abnormal (0/2)
Additional findings	Microcephaly (3/18)	Microcephaly (0/2)	Microcephaly (0/1)

Patient #1 is an 11-month-old girl, born at term with a birth weight of 3,250 g. Her parents were healthy and did not have a consanguineous relationship. She presented with febrile seizures at the age of 8 months. Generalized seizures occurred once a week and lasted for 2–3 min. The EEG displayed bursts of high-amplitude spikes and sharp waves in the bifrontal and temporal regions. The brain MRI was normal. Seizures were relieved following treatment with sodium valproate (VPA) for 2 months. Her cognitive and motor developments were not affected at the last follow-up. She carried a *de novo* variant (c.719A > G, p. E240G) of *HCN1*, and this variant was absent from gnomAD, and the amino acid is evolutionarily conserved among different species. It is predicted to be tolerant and deleterious in SIFT and PROVEAN, respectively. The variant meets ACMG/AMP guidelines (PS2 + PM1 + PM2 + PP3) to be considered likely pathogenic.

Patient #2 is a 1-year-old girl who had seizures within 2 days after birth. She was born at term with a birth weight of 3,000 g. Her parents were healthy and non-consanguineous. Her growth parameters were normal. At the age of 2 months, seizures became more frequent and severe. Seizures evolved into tonic asymmetric seizures with prolonged cyanosis and occurred two to four times a day. At 3 months, she developed more frequent seizures that occurred 20–30 times/day triggered by fever. At 4 months, she suffered from dozens of episodes per day and uncontrollable recurrent status epilepticus. Her overall development was regressed. Seizures could be slightly relieved by nitrazepam (NZP) but were resistant to levetiracetam (LEV) and VPA. Her brain MRI was normal, but her cardiac color ultrasound displayed a ventricular septal defect. EEG showed abnormal background activity including diffuse 1.5–5 Hz mixed slow waves with low-medium amplitude and a small number of fast waves with low amplitude in the awake state. Interictal EEG showed a large number of sharp waves, with sharp slow waves appearing suddenly or continuously. During the 15-h video EEG recording, 11 seizures were monitored, of which 6 partial seizures started with a paroxysmal ictal discharge in the left temporal region, 2 partial seizures started in the Rolandic region, and 3 seizures started in the right temporal region (Figure 2A). Unfortunately, at the age of 1 year, she died of uncontrollable seizures. Her trio-based exome analysis identified a *de novo* variant in *HCN1* (c.1138A > T, p. I380F). The variant is absent from gnomAD, and the amino acid is evolutionarily conserved among different species. The pathogenic evaluations in SIFT and PROVEAN were damaging and deleterious, respectively. It is considered pathogenic under ACMG/AMP guidelines (PS1 + PS2 + PM1 + PM2 + PP3).

Patient #3 was a 2-year-old girl, born at term. Her parents were non-consanguineous without a family history. At the age of 3 months, she had her first generalized tonic seizure after a fever. Status epilepticus appeared twice a month and lasted for 20 min. Her development milestone has been delayed since the first seizure. Her brain MRI was normal. The EEG showed multifocal spike waves in the right temporal and occipital and the left posterior regions (Figure 2B). No seizures were monitored during a 15-h video EEG recording. At first, she was treated with VPA, which improved her daily seizures. However, focal seizures and status epilepticus still appeared approximately twice a month. At the age of 17 months, she was absent of language and could not sit alone. Accordingly, oxcarbazepine (OXC) and lamotrigine (LTG) were added to the treatment but were ineffective. At the age of 20 months, clobazam (CLB) was added to her treatment. At the last follow-up, she had been seizure-free for 6 months and acquired slight improvements in language and motor development. Her trio-based exome analysis identified a *de novo* variant in *HCN1* (c.1184C > G, p. A395G). The variant is absent from gnomAD, and the amino acid is evolutionarily conserved among different species. The pathogenic evaluations in SIFT and PROVEAN were damaging and deleterious, respectively. The variant meets ACMG/AMP guidelines to be considered likely pathogenic (PS2 + PM1 + PM2 + PP3).

Patient #4 was a 2-year-old girl, who was born at term. Her parents were healthy and did not have a consanguineous relationship. She suffered from episodic paralysis, and could not walk at the age of 7 months. Three months later, she had seizures 2–3 times/day in addition to the paralysis. She did not show any prominent delay in motor and cognition development. Her brain MRI was normal. Four-hour video EEG showed a slow background, and diffusive fast waves in the occipital regions (Figure 2C). Fortunately, she became seizure-free with OXC treatment and got recovered from paralysis. Also, the 4-h video EEG was normal after the 6-month treatment of OXC. Exome sequencing analysis identified a *de novo* frame-shift variant in *HCN1* (c.2128-2129dup, p. S710Rfs*71). The variant is absent from gnomAD, and the amino acid is evolutionarily conserved among different species. Combined with the functional experiment in this study, it is considered a variant of pathogenic under ACMG/AMP guidelines (PVS1 + PS2 + PM2).

Patient #5 was a 7-year-old boy, who was born at term to a non-consanguineous family. His parents were healthy without a family history. He underwent normal development until the appearance of seizures at the age of 16 months. At first, he presented with febrile generalized tonic-clonic seizures. At the age of 3 years, he exhibited tonic-clonic seizures once a month with or without fever. His seizures were not well controlled with a combination therapy of VPA and OXC. LEV was added to the therapy at the age of 4 years, and he became seizure-free. However, he still presented with ataxia, tremor, slight language disorders, and motor disorders. There was a malformation in the right tragus. His brain MRI was normal. The EEG background displayed 4 Hz activity in the occipital region. No ictal discharge was monitored in the interictal EEG. EEG showed 3.5–4 Hz spike-wave discharges lasting for 90 s, while the patient was having a tonic-clonic seizure in sleep (Figure 2D). Exome sequencing analysis identified a variant in *HCN1* (c.1715T > C, p. V572A) inherited from his asymptomatic father. The variant is absent from gnomAD, and the amino acid is evolutionarily conserved among different species. No other pathogenic or likely pathogenic variants were detected accounting for his phenotypes. It was predicted to be damaging and deleterious both in SIFT and PROVEAN. It is considered a variant of uncertain significance under ACMG/AMP criteria (PVS1 + PS2 + PM2), while the subsequent functional experiment proved to be likely pathogenic.

### Biophysical Properties of Mutant Hyperpolarization-Activated Cyclic Nucleotide-Gated Ion Channel 1 Channels Expressed in HEK293 Cells

**FIGURE 3:**
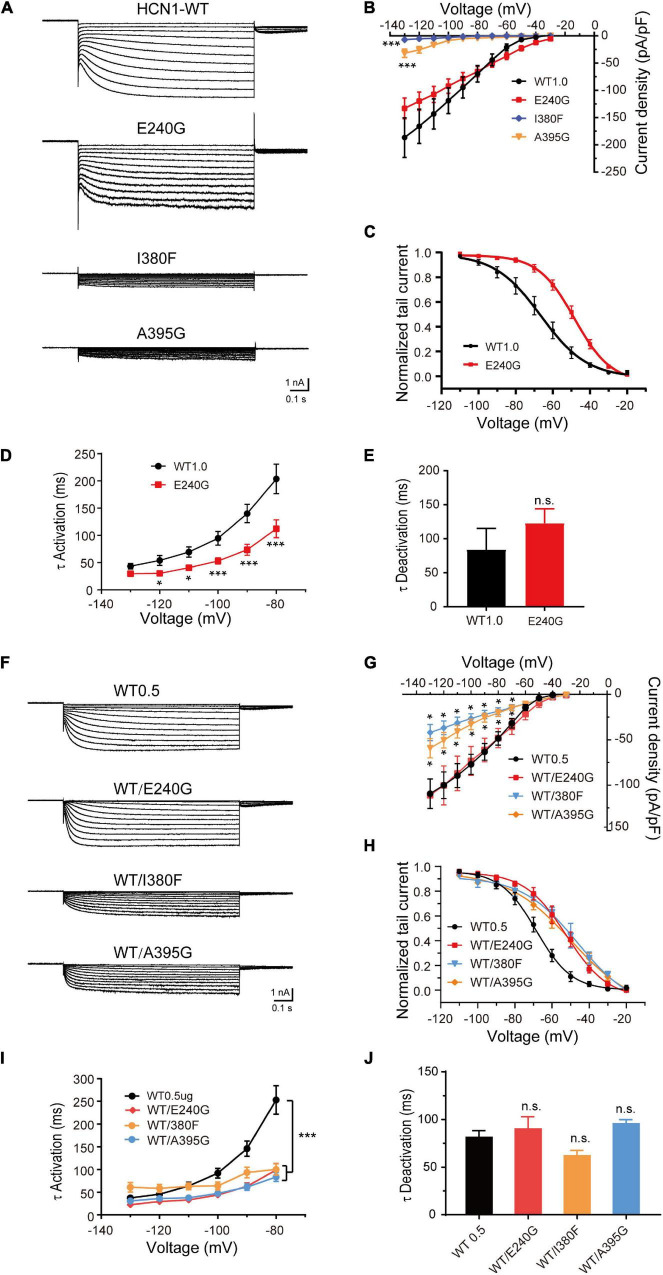
Electrophysiological properties of novel and *de novo* variants in *HCN1* located in or close to transmembrane regions. **(A)** Representative recordings of currents mediated by mutant HCN1 channels. **(B)** Current densities of E240G (*n* = 16), I380F (*n* = 9), and A395G channels (*n* = 8) (WT1.0: *n* = 9). Dunnett’s T3 *post hoc* test. **(C)** Activation curves of WT and E240G channels (WT: *n* = 12; E240G: *n* = 11), and lines represent Boltzmann functions fit to the data points. **(D)** Mean time constants of activation obtained for HCN-WT (black) and E240G channels (red). (WT: *n* = 8; E240G: *n* = 15). Dunnett’s T3 *post hoc* test. **(E)** Bar graph shows mean deactivation time constants of WT and E240G channels recorded at + 10 mV (WT: *n* = 8; E240G: *n* = 12). Two-sample *t*-test. **(F)** Representative traces of heterozygous channels. **(G)** Plot of mean current densities of WT/E240G (*n* = 13), WT/I380F (*n* = 13), WT/A395G (*n* = 12), and WT channels (*n* = 18). Dunnett’s T3 *post hoc* test. **(H)** Activation curves of WT channels (*n* = 6, black), WT/I380F channels (*n* = 6, blue), WT/E240G (*n* = 11, red), and WT/A395G channels (*n* = 12, orange). Bonferroni *post hoc* test. **(I,J)** Graphs show the effects of the activation (WT/E240G: *n* = 17; WT/I380F: *n* = 12; WT/A395G: *n* = 19; WT0.5: *n* = 12) and deactivation time constants (WT/I380F: *n* = 12; WT/E240G: *n* = 17; WT/A395G *n* = 17; WT0.5 *n* = 14) induced by mutant heterozygous channels. Bonferroni *post hoc* test. All data are presented as mean ± S.E.M. values (n.s. = not significant, *P < 0.05, ***P < 0.005, in this and other figures).

To verify the effects of variants on functional properties of HCN1 channels, we transfected WT, mutant, and WT/mutant constructs into HEK cells. Representative traces of currents mediated by HCN1 channels were recorded from HEK cells expressing different mutant channels (Figures 3A,F). The E240G mutant did not affect current densities (WT: –186.7 ± 36.28 pA/pF; E240G: –144 ± 19.46 pA/pF; Dunnett’s T3 test, *P* > 0.05), but induced a rightward shift in the activation curve of 19 mV (Figures 3A–C). So, we recognized this variant produced a gain-of-function effect, and the activation time constant (τ) was significantly shorter than that of WT channels within the range of –80 mV to –130 mV (Dunnett’s T3 test, *P* < 0.05; Figure 3D) with deactivation time constants not affected (Two-sample *t*-test; *P* > 0.05; Figure 3E). The current densities of I380F and A395G were much smaller than WT channels at –130 mV (I380F: –7.14 ± 2.20 pA/pF; A395G: –31.95 ± 7.84 pA/pF) (Figures 3A,B), which indicated that these two variants likely exerted severe loss-of-function effects on HCN1 channels.

To investigate the function of heterozygous HCN1 channels formed by WT and mutant subunits, we performed co-expression experiments. WT/E240G channels produced a depolarizing shift in the activation curve of 15.9 mV (Bonferroni *post hoc* test, *P* < 0.05; Figures 3F,H), and the activation time constants were faster than the WT channels (Bonferroni *post hoc* test, *P* < 0.05; Figures 3I,J), which was similar to the homozygous state. Both heterozygous I380F channels and A395G channels presented with smaller current densities (Dunnet T3 test, *P* < 0.05; Figures 3F,G) and depolarized the activation curves by 24.4 and 18.1 mV, respectively (Bonferroni *post hoc* test, *P* < 0.05; Figure 3H). The activation time constants (–80 mV) were reduced by heterozygous WT/A395G channels (WT = 253.10 ± 31.37 ms; WT/A395G = 83.70 ± 9.84 ms; Bonferroni *post hoc* test, *P* < 0.05) and WT/I380F (Bonferroni *post hoc* test, WT/I380F = 100.03 ± 12.71 ms; Bonferroni *post hoc* test, *P* < 0.05) (Figure 3I). The deactivation time constants of heterozygous variant channels did not show any significant changes (WT1.0: 83.97 ± 31.24 ms; WT/I380F: 61.86 ± 5.70 ms; WT/A395G: 95.65 ± 4.25 ms; Bonferroni *post hoc* test, *P* > 0.05) (Figure 3J). These results indicate variants produced negative effects on the function of HCN1 channels in the heterozygous conditions.

**FIGURE 4:**
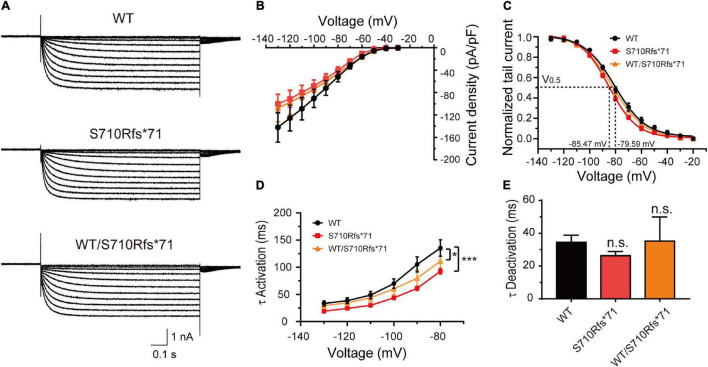
Functional changes of the S710Rfs*71 variant. **(A)** Representative recordings of currents mediated by HCN1 channels. **(B)** Plot of mean current densities (pA/pF) of WT, S710Rfs*71, and WT/S710Rf*71 channels (WT: *n* = 14; S710Rfs*71: *n* = 20; WT/S710Rfs*71: *n* = 16). **(C)** Activation curves obtained by normalizing the tail currents of WT, S710Rfs*71, and WT/S710Rf*71 channels (WT: *n* = 11; S710Rfs*71: *n* = 13; WT/S710Rf*71: *n* = 7). **(D)** Plot of mean activation time constants obtained for HCN1-WT, S710Rfs*71, and WT/S710Rf*71 channels (WT: *n* = 16; S710Rfs*71: *n* = 16; WT/S710Rf*71: *n* = 6). **(E)** Bar graph shows mean deactivation time constants obtained for HCN1-WT, S710Rfs*71, and WT/S710Rf*71 channels (WT: *n* = 12; S710Rfs*71: *n* = 16; WT/S710Rfs*71: *n* = 6). All the statistical analyses in this figure were performed by one-way ANOVA and Bonferroni *post hoc* test. *P < 0.05, ***P < 0.005.

Given that the C-terminal region is important to the activation kinetics, we investigated whether S710Rfs*71 or V572A affected channel function in order to assess the pathogenicity of these variants. The half-activation voltage of S710Rfs*71 was shifted to the left by 5.88 mV (Bonferroni *post hoc* test, *P* < 0.05), and the activation time constants were faster than those of WT channels (–130 mV: WT, 33.41 ± 4.60 ms; S710Rfs*71, 19.10 ± 1.06 ms; *P* < 0.05). The other properties of HCN1 channels including current density, activation curve, and the deactivation time constants were not affected. Co-expression experiments indicated that the activation time constants of WT/S710Rfs*71 were slightly faster than the WT channels (–130 mV: S710Rfs*71, 29.41 ± 5.42 ms; *P* < 0.01). However, current densities, activation curve, and deactivation time constants of WT/S710Rfs*71 were not affected (Figure 4).

**FIGURE 5:**
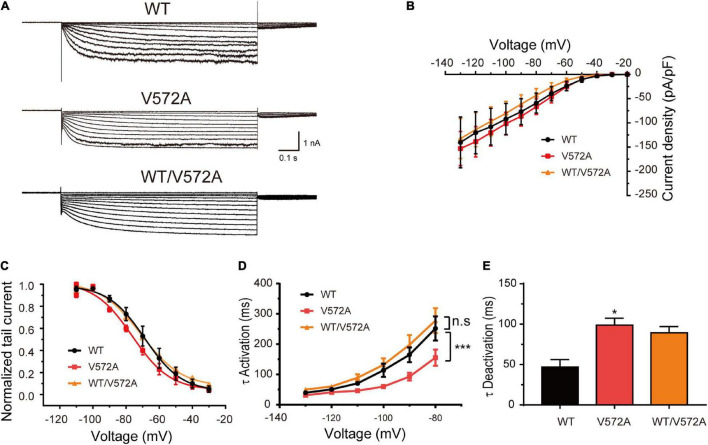
Functional changes of the V572A variant. **(A)** Representative traces of WT, V572A, and WT/V572A channels. **(B)** Plot of mean current densities of WT (*n* = 11), V572A (*n* = 10), and WT/572A channels (*n* = 14). Two-sample *t*-test. **(C)** Voltage-dependent activation curves of WT channels (*n* = 9), V572A (*n* = 11), and WT/572A channels (*n* = 8). Analysis was done by two-sample *t*-test. **(D,E)** Summaries of mean activation time constants (WT: *n* = 16; V572A: *n* = 8; WT/V572A *n* = 11) and deactivation constants (WT: *n* = 10; V572A: *n* = 9; WT/V572A *n* = 12) for WT, V572A, and WT/572A. Analysis was done by two-sample *t*-test. *P < 0.05, ***P < 0.005.

In addition, the current densities and activation curves of V572A channels were unaffected in comparison with WT channels (Two-sample *t*-test, *P* > 0.05). However, V572A channels exhibited faster activation (at –110 mV: WT, 70.50 ± 6.98 ms; V572A, 46.19 ± 3.82 ms; two-sample *t*-test, *P* < 0.05) and slower deactivation (WT: 46.89 ± 9.18 ms; V572A: 98.76 ± 6.06 ms; two-sample *t*-test, *P* < 0.05) (Figure 5). The biophysical properties of WT/V572A channels were not changed compared to those of WT channels. These data demonstrated that variants affecting the C-terminal region also impaired channel function, including activation and deactivation kinetics.

### The Expression of Hyperpolarization-Activated Cyclic Nucleotide-Gated Ion Channel 1 Channels in Neurons Was Impaired by Hyperpolarization-Activated Cyclic Nucleotide-Gated Ion Channel 1 Variants

**FIGURE 6:**
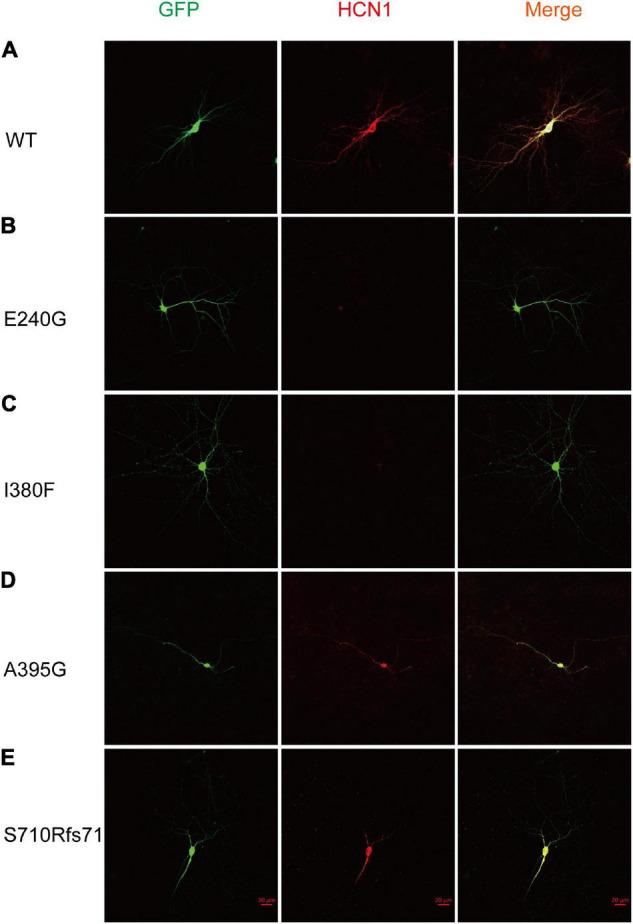
Mutant HCN1 channels distributed differently in cortical neurons. **(A–E)** The expression of WT (*n* = 13), E240G (*n* = 7), I380F (*n* = 9), A395G (*n* = 9), and S710Rfs*71 (*n* = 9) channels in cortical neurons, respectively.

To estimate the expression levels of HCN1 protein, we transfected WT and mutant plasmids into cortical neurons. In accordance with previous studies (Noam et al., 2010), immunoreactive HCN1 channels were detected in cortical neurons transfected with WT plasmids. The expressing patterns of A395G and S710Rfs71 channels were similar to that of WT channels. However, we observed that neurons expressing E240G and I380F channels showed weak immunoreactive HCN1 channels (Figure 6). These observations indicated that variants could reduce HCN1 protein expression in neurons.

### Impact of Hyperpolarization-Activated Cyclic Nucleotide-Gated Ion Channel 1 Variants on Neuronal Excitability

**FIGURE 7:**
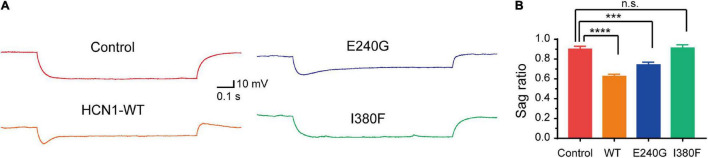
Hyperpolarizing voltage sag in cortical neurons expressing WT and mutant channels. **(A)** Representative recordings of voltage sag in response to a −100 pA hyperpolarizing current injection in WT and mutant *HCN1*-transfected neurons. **(B)** Bar graph shows mean voltage sag ratios for different types of HCN1 channels. ***P < 0.005, ****P < 0.001.

Given that HCN1 channels mediate non-selective cation currents which play critical roles in neuronal excitability, we further studied the effects of *HCN1* variants on neurons. We chose two *HCN1* variants (E240 and I380F) with different biophysical properties in HEK293 cells. We observed neurons expressing WT, and E240G channels displayed voltage sags in response to a hyperpolarizing step (Figure 7). As the I380F channels lost the ability to mediate currents, the voltage sag was absent in neurons expressing the I380F channel (Figure 7).

**FIGURE 8:**
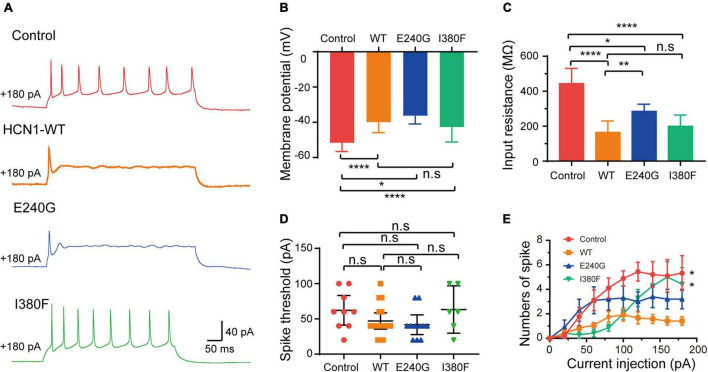
Neuronal excitability was affected by HCN1 variants. **(A)** Representative recordings of action potential firing patterns in response to current injection (180 pA). **(B)** Bar graph summarizes the mean membrane potential in neurons expressing different HCN1 channel types. Analysis was done by Bonferroni *post hoc* test. **(C)** Bar graph shows mean input resistance in neurons expressing different HCN1 channel types. Dunnett’s T3 *post hoc* test. **(D)** Bar graph shows spike thresholds elicited by current injection were not affected. Analysis was done by Mann–Whitney test. **(E)** Plot of the mean firing rates of neurons in response to current injections. *P < 0.05, **P < 0.01, ****P < 0.001.

As expected from the biophysical properties of HCN channels, neurons transfected with all types of HCN1 channels resulted in a depolarization of the membrane potential of neurons compared to the neurons transfected with plasmid vectors (Figure 8B) (Control: –51.7 ± 1.75 mV; WT: –40 ± 1.9 mV; E240G: –36.3 ± 1.46 mV; I380F: –42.80 ± 3.20 mV). Neurons transfected with all types of channels showed decreased input resistance (Control: 443.5 ± 30.87 MΩ; WT: 164.1 ± 17.12 MΩ; E240G: 284.7 ± 43 MΩ; I380F: 299.5 ± 26.3 MΩ). Both WT/E240G and WT/I380F caused a right shift in the activation curves, which indicated HCN1 channels were more likely to open at more depolarized potential, thus affecting resting membrane potential as well as input membrane resistance (Figure 8C). However, E240G and I380F channels might affect neuronal activity differently, since the two channels differ in biophysical properties. Considering this issue, we compared the excitability of neurons expressing WT or mutant channels under depolarizing current injection. Spike thresholds of neurons were not affected by either WT or mutant channels (Figure 8D). Neurons expressing WT and E240G channels showed a reduction in the number of action potentials fired in response to depolarizing current steps compared to those expressing empty channels, while neurons transfected with *HCN1* I380F showed a higher firing rate compared to the neurons expressing WT channels (Figures 8A,E). These data indicated mutant channels with different biophysical properties might exert different effects on neuronal excitability.

## Discussion

In the present study, we identified four novel variants causing *HCN1*-related epilepsy and investigated functional changes of the five variants in five new cases. According to our study and a previous study (Marini et al., 2018), the clinical phenotypes caused by different mutations overlap in many aspects, including the early age of onset, febrile sensitivity, seizure types, and developmental delay. The electrophysiological experiments verified functional changes of the five variants. In addition, two variants (E240G and I380F) showed reduced protein expression in the cortical neurons of mice and affected neuronal excitability.

The dysfunctions of the mutant HCN1 channels, including changes in current densities and activation/deactivation kinetics, played essential roles in neuronal excitability. The E240G mutation produced a gain-of-function effect due to the right-shift activation curve and faster activation time constants. The I380F and A395G mutations significantly reduced current densities, suggesting a loss-of-function effect. The S710Rfs*71 mutation showed a loss- and gain-of-function effect since the activation curve shifted to the left and the activation time constants were reduced. The V572 mutation displayed a faster activation, suggesting a GOF effect. Besides, all the three *HCN1* mutations (E240G, I380F, and A395G) produced dominant-negative effects in heterozygous conditions. Notably, some variants might cause some confusing effects in heterozygous conditions. For example, two LOF mutations (I380F and A395G) displayed a significant rightward shift in the activation curve in heterozygous conditions implying a GOF effect, which is similar to the previously reported mutation (M305L).

Also, when the variants were expressed in neurons, they exerted dominate-negative effects, since a mutant subunit could be assembled into heteromeric channels with WT subunits. Epilepsy has been recognized as a brain disorder resulting from neuronal hyperexcitability and hypersynchronous firing. In addition to the findings that LOF and GOF *HCN1* mutants could cause epilepsy, we unveiled that the *HCN1* mutants could exert effects on neurons in different manners. First, we found that the protein expression of HCN1 channels in neurons could also be affected. The surface expression of HCN1 depends on many factors, including protein synthesis and trafficking. Interestingly, we noted that these two variants caused more severe clinical phenotypes than other mutants, as patients presented with more frequent seizures, longer seizure duration, and even sudden death. Although altered protein expression might not be sufficient to clarify the phenotypes, it is advisable to pay more attention to the protein expression in different neurons when evaluating the pathogenicity of the mutants. Second, LOF and GOF variants may impose effects on neural excitability differently. The present study showed that neurons transfected with *HCN1* E240G might contribute to neuronal hyperexcitability by depolarizing membrane potential and impairing the ability to fire action potentials. Since the reversal potential of HCN1 channels is higher than the threshold for action potential generation, HCN1 channel currents depolarize the membrane potential when it reaches the resting membrane potential (Robinson and Siegelbaum, 2003). Besides, firing deficits of excitatory neurons might impair information transmission among neurons. Accordingly, this epileptic activity caused by GOF mutants could be reduced by HCN1 channel inhibitors (Inaba et al., 2006).

It is worth noting that the *HCN1* I380F variant increased firing rate, which suggests LOF variants could cause hyperexcitability of excitatory neurons. Previous studies have shown that two LOF variants (L157V and M294L) depolarized membrane potential, decreased input resistance, and increased firing rate (Bonzanni et al., 2018; Bleakley et al., 2021), thus contributing to neuronal hyperexcitability. *HCN1* null mice showed higher susceptibility to seizures and higher mortality rates (Santoro et al., 2010). Additionally, dendrites could become more excitable due to the enhancement of excitatory synaptic inputs that impair the excitatory/inhibitory balance in neuronal circuits following a reduction in input resistance (Huang et al., 2009; Yi et al., 2016). It is notable that seizures, particularly for status epilepticus, decrease the expression and suppress the function of HCN1 channels (Shin et al., 2008), which may imply worse endings of epilepsy caused by LOF variants.

Previous studies have shown that the biophysical properties of channelopathy-causing variants, such as *SCN1A* (Berecki et al., 2019), *KCNA2* (Syrbe et al., 2015), *KCNQ2* (Miceli et al., 2015), and *GluN2B* (Platzer et al., 2017) may correlate with phenotypes. By analyzing the results of functional experiments of 17 variants in previous studies and five variants in our study, we found that LOF variants caused more severe and complicated phenotypes (Table 1 and Supplementary Table 2). We observed that LOF variants displayed a higher rate of epilepsy onset within the first year of life (13/18), death (3/18), status epilepticus (5/18), uncontrolled seizures (10/18), and moderate/severe intellectual disabilities (12/18). We also observed that LOF variants might be more likely to cause microencephaly (3/18) and abnormalities in brain structure (3/14), while no patient carrying GOF variants presented with microencephaly or abnormal neuroimaging. However, it is difficult to distinguish the types of variants according to phenotypes, because phenotypes of patients with LOF variants or GOF variants overlap in some aspects, such as the age of seizure onset, seizure types, intellectual disorders, and EEG. Additionally, we noted six out of seven variants located in the S5 through S6 domains showed LOF actions by reducing the current densities of the channels, and four out of seven variants located in the C-terminal domain displayed GOF actions by shifting the voltage-dependent activation curves to the right. Coincidentally, a previous study (Marini et al., 2018) showed that variants located in transmembrane or the domains of pore structure (from S5 to S6) caused more severe phenotypes compared to those located in the C-terminal region. It is known that a loop between the S5 and S6 domains, forming an ion selectivity filter and a C-terminal domain, affects the voltage dependence of activation (Postea and Biel, 2011; Lee and MacKinnon, 2017). Despite the fact that both biophysical properties and the location of variants may correlate with the severity of phenotypes, and the location of mutant residue determines the channel function to some extent, we are unable to determine which factor plays a decisive role. In the future, more functional studies are necessary to verify this.

## Conclusion

In conclusion, the present study expands the genotypic and phenotypic spectrum of patients with HCN1-related epilepsy and clarifies the underlying mechanisms. We reported five new cases, including four unreported pathogenic/likely pathogenic variants. We provided an assessment of biophysical function for each variant, which could help patients to receive individual therapy in the future. We confirmed that *HCN1* variants contributed to neural hyperexcitability by regulating input resistance and firing rate of action potentials, and we have shown that they can affect protein expression in neurons for the first time.

## Data Availability Statement

The datasets presented in this study can be found in online repositories. The names of the repository/repositories and accession number(s) can be found in the article/Supplementary Material.

## Ethics Statement

The studies involving human participants were reviewed and approved by the ethics committee of Xiangya Hospital of Central South University. Written informed consent to participate in this study was provided by the participants’ legal guardian/next of kin. The animal study was reviewed and approved by the ethics committee of Xiangya Hospital of Central South University. Written informed consent was obtained from the individual(s), and minor(s)’ legal guardian/next of kin, for the publication of any potentially identifiable images or data included in this article.

## Author Contributions

CX and JP conceived the experiments and wrote the article. CX and FL performed functional experiments and analyses. FH, XW, LM, HH, and FY carried out genetic analyses of patients. JP analyzed seizure types of patients and carried out clinical characterization. All authors contributed to the article and approved the submitted version.

## Conflict of Interest

The authors declare that the research was conducted in the absence of any commercial or financial relationships that could be construed as a potential conflict of interest.

## Publisher’s Note

All claims expressed in this article are solely those of the authors and do not necessarily represent those of their affiliated organizations, or those of the publisher, the editors and the reviewers. Any product that may be evaluated in this article, or claim that may be made by its manufacturer, is not guaranteed or endorsed by the publisher.

## Footnotes

1
http://gnomad.broadinstitute.org/
