# Supplementary material for: Novel HCN1 Mutations Associated With Epilepsy and Impacts on Neuronal Excitability
Source: Front Mol Neurosci. 2022 Jun 30;15:870182. doi: 10.3389/fnmol.2022.870182 (PMC9280081; doi:10.3389/fnmol.2022.870182)
Supplement: Supplementary file 1 [file Data_Sheet_1.PDF]

Supplementary Figures

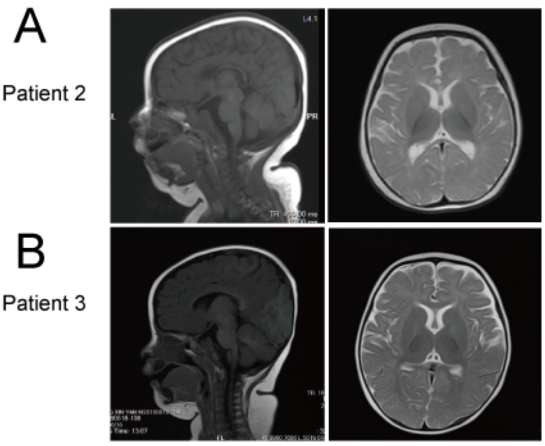

Supplementary Fig. 1 MRI of two patients carrying *HCN1* variants.

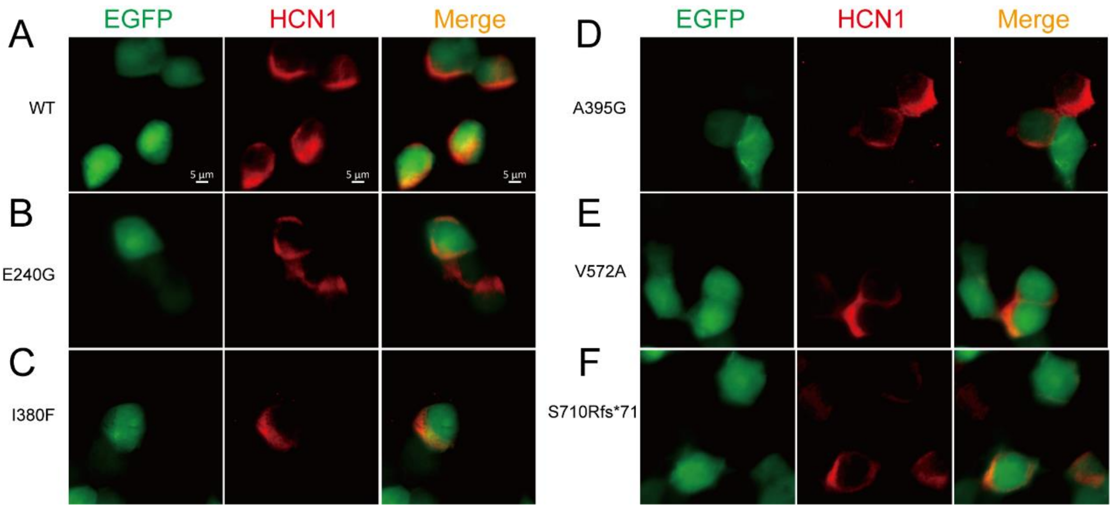

Supplementary Fig. 2 Fluorescence images of WT and mutant constructs transfected in HEK293 cells. As indicated, each row shows a different HCN1 channels. Columns from left to right show HCN1 channels (red), EGFP (green) and merged images. N = 8-10 for each group.

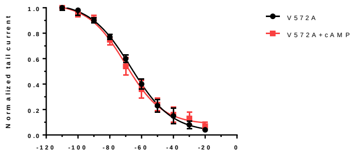

Supplementary Fig. 3 cAMP had no effect on activation curve of the variant of V572A.
